# Computational detection of a genome instability‐derived lncRNA signature for predicting the clinical outcome of lung adenocarcinoma

**DOI:** 10.1002/cam4.4471

**Published:** 2021-12-05

**Authors:** Chen‐Rui Guo, Yan Mao, Feng Jiang, Chen‐Xia Juan, Guo‐Ping Zhou, Ning Li

**Affiliations:** ^1^ Department of Abdominal Oncology The Second Affiliated Hospital of Zunyi Medical University Zunyi China; ^2^ Department of Pediatrics The First Affiliated Hospital of Nanjing Medical University Nanjing China; ^3^ Department of Neonatology, Obstetrics and Gynecology Hospital of Fudan University Shanghai China; ^4^ Department of Nephrology Affiliated Hospital of Nanjing University of Chinese Medicine, Jiangsu Province Hospital of Chinese Medicine Nanjing China

**Keywords:** genome instability, long non‐coding RNAs, lung adenocarcinoma, mutator phenotype

## Abstract

Evidence has been emerging of the importance of long non‐coding RNAs (lncRNAs) in genome instability. However, no study has established how to classify such lncRNAs linked to genomic instability, and whether that connection poses a therapeutic significance. Here, we established a computational frame derived from mutator hypothesis by combining profiles of lncRNA expression and those of somatic mutations in a tumor genome, and identified 185 candidate lncRNAs associated with genomic instability in lung adenocarcinoma (LUAD). Through further studies, we established a six lncRNA‐based signature, which assigned patients to the high‐ and low‐risk groups with different prognosis. Further validation of this signature was performed in a number of separate cohorts of LUAD patients. In addition, the signature was found closely linked to genomic mutation rates in patients, indicating it could be a useful way to quantify genomic instability. In summary, this research offered a novel method by through which more studies may explore the function of lncRNAs and presented a possible new way for detecting biomarkers associated with genomic instability in cancers.

## Graphical abstract

The emerging proof of long non‐coding RNAs (lncRNAs) as important components of genome instability have been revealed. Here, we established a six lncRNA‐based signature, which was found closely linked to genomic mutation rates in patients, indicating it could be a powerful way to quantify genomic instability.

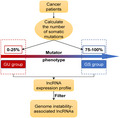

## 1 INTRODUCTION

The most prevalent source of cancer‐associated mortality worldwide is lung cancer, and the most common histological type is lung adenocarcinoma (LUAD), accounting for approximately half of the cases.^1^, ^2^, ^3^ LUAD is associated with high degree of malignancy and a poor prognosis.^4^, ^5^ The therapy for LUAD is based on the grade and stage, which is primarily defined by the assessment of tumor histology and patient characteristics by pathologists.^6^ Clinical features of patients (such as age, gender, stage, etc.) are currently commonly used prognostic factors of LUAD.^7^ However, it is acknowledged that LUAD is a complex disease characterized by genetic, clinical, and pathological heterogeneity. For instead, LUAD shows molecular characteristics that vary according to the patients’ smoking history.^8^ Therefore, to better determine the clinical results of patients with LUAD, better prognostic biomarkers are needed.

Genomic instability has shown to be a marker of prognosis, and it is correlated with tumor proliferation and survival.^9^, ^10^ It has been proposed that the molecular foundation of genomic instability remains unclear. Also, abnormal transcriptional and post‐transcriptional activities have been reported linked to genomic instability,^11^ which indicates the possibility of a molecular signature to serve as the quantitative indicator of genomic instability. For instance, Bao et al.^12^ examined 795 specimens of breast cancer and found 128 novel long non‐coding RNAs (lncRNAs) that were correlated with genomic instability. The Wang's study established a 10‐miRNA‐based signature related to genomic instability in ovarian cancer.^13^, ^14^ LncRNAs are transcripts that are greater than 200 nucleotides that do not have coding ability.^15^ Numerous studies over the past few years have shown that lncRNAs play a role in various biological functions,^16^, ^17^ especially that the abnormal expressions of lncRNAs may have a significant impact on cancer progression, including proliferation, migration, invasion and cancer metastasis.^18^ Several lncRNAs have been found expressed in tumor tissues, such as MALAT1^19^ and H19.^20^ The utilization of next‐generation sequencing technologies to examine expression profiles of a significant number of lncRNAs has opened new possibilities for the assessment of the role of lncRNAs.^21^, ^22^, ^23^ Recent literature has shown that lncRNAs perform crucial roles in the preservation of genomic instability.^24^ A recent research has shown a particular lncRNA, NORAD, contributing to genomic stability by interacting with proteins involved in the process of DNA replication and repair.^25^ CUPID1 and CUPID2, two human lncRNAs recently found by Betts et al., regulate the DNA repair‐associated genes.^26^ Another lncRNA, named DDSR1, is thought to be essential in genomic instability through interacting with proteins involved in the process of DNA damage and controlling the expression levels of corresponding genes.^27^, ^28^ Despite some lncRNAs have been found to participate in maintaining genomic stability, the therapeutic importance of them in cancer is still mainly unexplored.

In this research, to assess the possibility of lncRNA‐based signature to serve as a predictor of genomic instability, we tried to build a computational frame derived from mutator hypothesis. The frame combined the profiles of lncRNA expression with those of somatic mutations in a tumor genome, thus enhancing its prognostic function.

## 2 MATERIALS AND METHODS

### 2.1 Information review

The details regarding clinical characteristics, RNA‐seq expression, and somatic mutations data of LUAD samples were obtained from the database online, which is called The Cancer Genome Atlas (TCGA). The lncRNA expression details is collected from the TANRIC database.^29^ For further analysis, 490 samples were retained, including survival details, somatic mutation information, lncRNA and mRNA expression information, and typical clinical features. All of the patients with LUAD used were assigned into two sets. The set named the training set consisted of 246 patients, and was used to define the lncRNA‐based signature with prognostic value and develop the prognostic risk model. The other set named testing set consisted of 244 patients and was used to objectively verify the efficacy of the signature. The predictive value of the prognostic signature was then further investigated in the whole TCGA set. Other three separate LUAD sets named GSE68465,^29^ GSE10072^30^ and GSE30219^31^ were acquired from another database (the Gene Expression Omnibus, GEO) for further confirmation. The clinical and pathological characteristics of LUAD patients in the TCGA database were briefly addressed in Table 1.

**TABLE 1:** Information of clinical features in the three LUAD sets from TCGA

Covariates	Type	TCGA set (*n* = 490)	Testing set (*n* = 244)	Training set (*n* = 246)	*p*‐value
Age, no (%)	<=65	231 (47.14%)	119 (48.77%)	112 (45.53%)	0.3682^a^
	>65	249 (50.82%)	117 (47.95%)	132 (53.66%)	
	Unknow	10 (2.04%)	8 (3.28%)	2 (0.81%)	
Gender, no (%)	Female	262 (53.47%)	129 (52.87%)	133 (54.07%)	0.8612^a^
	Male	228 (46.53%)	115 (47.13%)	113 (45.93%)	
Stage, no (%)	Stage I–II	378 (77.14%)	184 (75.41%)	194 (78.86%)	0.179^a^
	Stage III–IV	104 (21.22%)	59 (24.18%)	45 (18.29%)	
	Unknow	8 (1.63%)	1 (0.41%)	7 (2.85%)	
T, no (%)	T1‐2	426 (86.94%)	210 (86.07%)	216 (87.8%)	0.745^a^
	T3‐4	61 (12.45%)	32 (13.11%)	29 (11.79%)	
	Unknow	3 (0.61%)	2 (0.82%)	1 (0.41%)	
M, no (%)	M0	324 (66.12%)	159 (65.16%)	165 (67.07%)	0.0649^a^
	M1	24 (4.9%)	17 (6.97%)	7 (2.85%)	
	Unknow	142 (28.98%)	68 (27.87%)	74 (30.08%)	
N, no (%)	N0	317 (64.69%)	160 (65.57%)	157 (63.82%)	0.7003^a^
	N1‐3	162 (33.06%)	78 (31.97%)	84 (34.15%)	
	Unknow	11 (2.24%)	6 (2.46%)	5 (2.03%)	

^a^ Chi square test.

### 2.2 Detection of genomic instability‐associated lncRNAs

**FIGURE 1:**
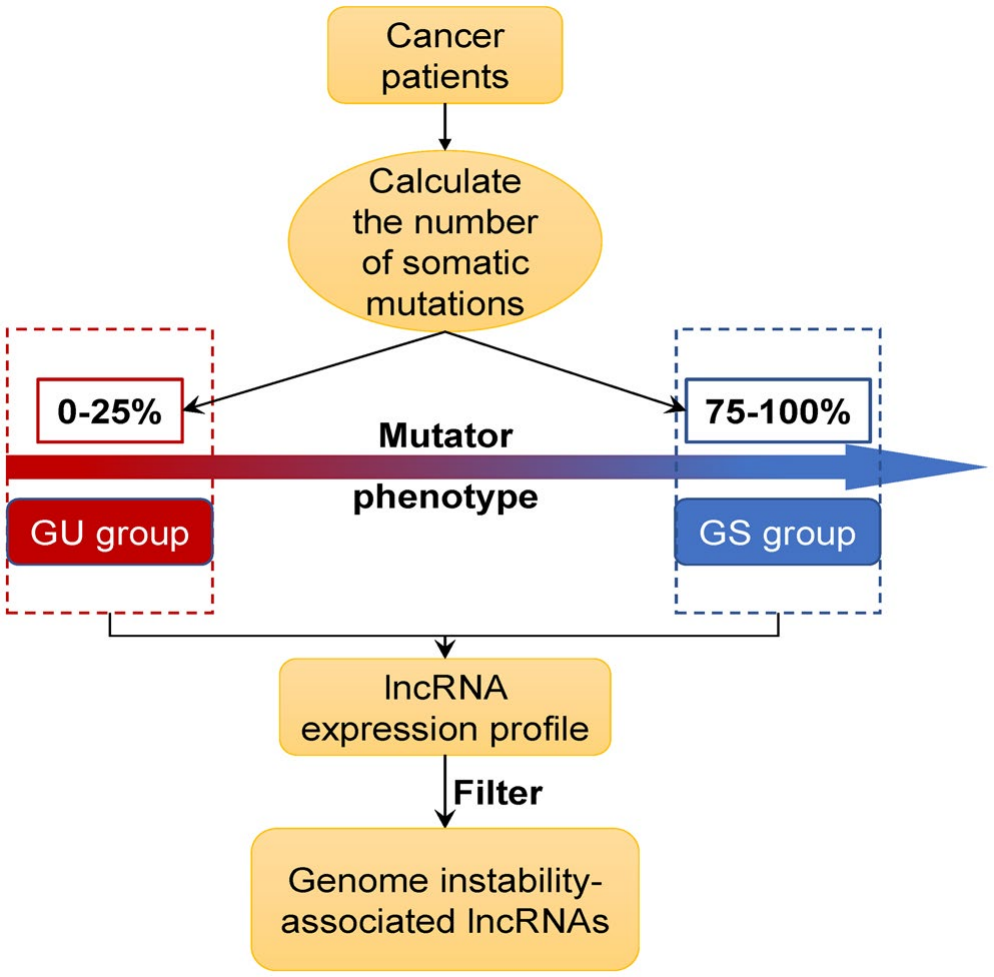
Computational description of identifying lncRNAs linked to genomic instability. We developed a somatic mutation profile. For each patient, the total number ofS somatic mutations was estimated. The numbers were then sorted in a decreasing order. Next, LUAD patients were classified into two groups, including the GU‐like group (the top 25 percent) and the GS‐like group (the last 25 percent), on the basis of the mutator phenotype. Through analyzing the lncRNA expression profiles between the two groups, lncRNAs, which had a significant correlation with genomic instability, were discovered

After combining somatic mutation profiles and lncRNA expression profiles in a tumor genome, a computational frame derived from mutator hypothesis was used to classify genome instability‐associated lncRNAs. The frame was listed in Figure 1: (a) the total number of somatic mutations was calculated; (b) LUAD patients in the training set were listed in a decreasing order according to their somatic cumulative mutations; (c) the last quarter of patients in the training set were assigned into the genomic stable‐like group (GS‐like group), while the top quarter of them were assigned into the genomic unstable‐like group (GU‐like group); (d) Comparison of the lncRNAs’ expression profiles between the two groups was performed with significance analysis of microarrays (SAM) method; (e) lncRNAs differentially expressed in two groups were considered to be genomic instability‐associated lncRNAs (GILncRNAs).

### 2.3 Statistical analysis

Ward's linkage method and Euclidean distances method were used to conduct hierarchical cluster research, by which LUAD patients in the training set from TCGA were separated into two clusters, the GU‐like cluster and the GS‐like cluster with different somatic mutation counts. For constructing the prognostic signature from the 185 genomic instability‐associated lncRNAs, univariate Cox regression analysis was carried out in the training set to screen the genomic instability‐associated lncRNAs, whose expression levels were closely associated with overall survival (OS). Then, the lncRNAs, whose Cox P‐value were smaller than 0.05, were considered as hub ones with prognostic value, and were included in the multivariate Cox regression analysis. The variables with *p*‐value <0.05 in the multivariate Cox analysis were chosen as the optimal ones to construct the signature. Finally, on the basis of the coefficients from the multivariate regression analysis and the expression levels of those optimal lncRNAs with prognostic value, we built a genome instability‐derived lncRNA signature (GILncSig) for predicting the clinical outcome of LUAD patients. The GILncSig was as followed:

$$\text{GILncSig}\;\left(\text{patient}\right)\;=\;\sum_{i=1}^{n}\;\text{coef}\left(\text{lncRNAi}\right)*\;\text{expr}\left(\text{lncRNAi}\right).$$

In LUAD, the score of GILncSig represented the prognostic risk level of patients. In this signature, lncRNAi means the ith prognostic lncRNA. Coef(lncRNAi) referred to the coefficient of multivariate Cox regression analysis, which represented the lncRNAi's contribution to prognostic risk scores. And the expr(lncRNAi) represents the lncRNAi's expression level. In the training set, we used the median score of LUAD patients to be a risk cutoff, which classified the patients into the low‐ group or high‐risk group according to their GILncSig scores.

Kaplan–Meier method was carried out to assess the survival rate for the two groups with different prognostic risks. Using the log‐rank test, the difference in survival between the two risk groups with a meaningful amount of 5 percent was calculated. Multivariate Cox regression analysis and stratified analysis were carried out to evaluate the association between the GILncSig scores and other clinical features. Hazard ratios (HRs) were calculated using Cox analysis. The receiver operating characteristic (ROC) curve dependent on time was also used to evaluate the prognostic performance of the GILncSig. Statistical analyses were carried out with R‐software 4.0.3.^32^

### 2.4 Functional enrichment analysis

We measured the relationship between the expression of lncRNAs and paired mRNAs using Pearson correlation method. The top ten mRNAs were then classified as the co‐expressed partners of corresponding lncRNAs. Functional enrichment analyses of those co‐expressed partners were performed to identify the markedly enriched Kyoto Encyclopedia of Genes and Genomes (KEGG) pathways and Gene Ontology (GO) terms to further predict the lncRNAs’ potential functions. The functional enrichment analysis was conducted in R‐version 4.0.3 using clusterProfiler tools. The Gene Set Enrichment Analysis (GSEA) was also carried out to further investigate the function of this genomic instability lncRNA‐based signature, including its molecular function and gene‐gene network. GSEA was performed on the basis of the while TCGA set, where 1000 random sample permutations were carried out, with a significance threshold of FDR <0.1 and *p* < 0.05.

## 3 RESULTS

### 3.1 Identification of genomic instability‐related lncRNAs (GILncRNAs) in LUAD patients

**FIGURE 2:**
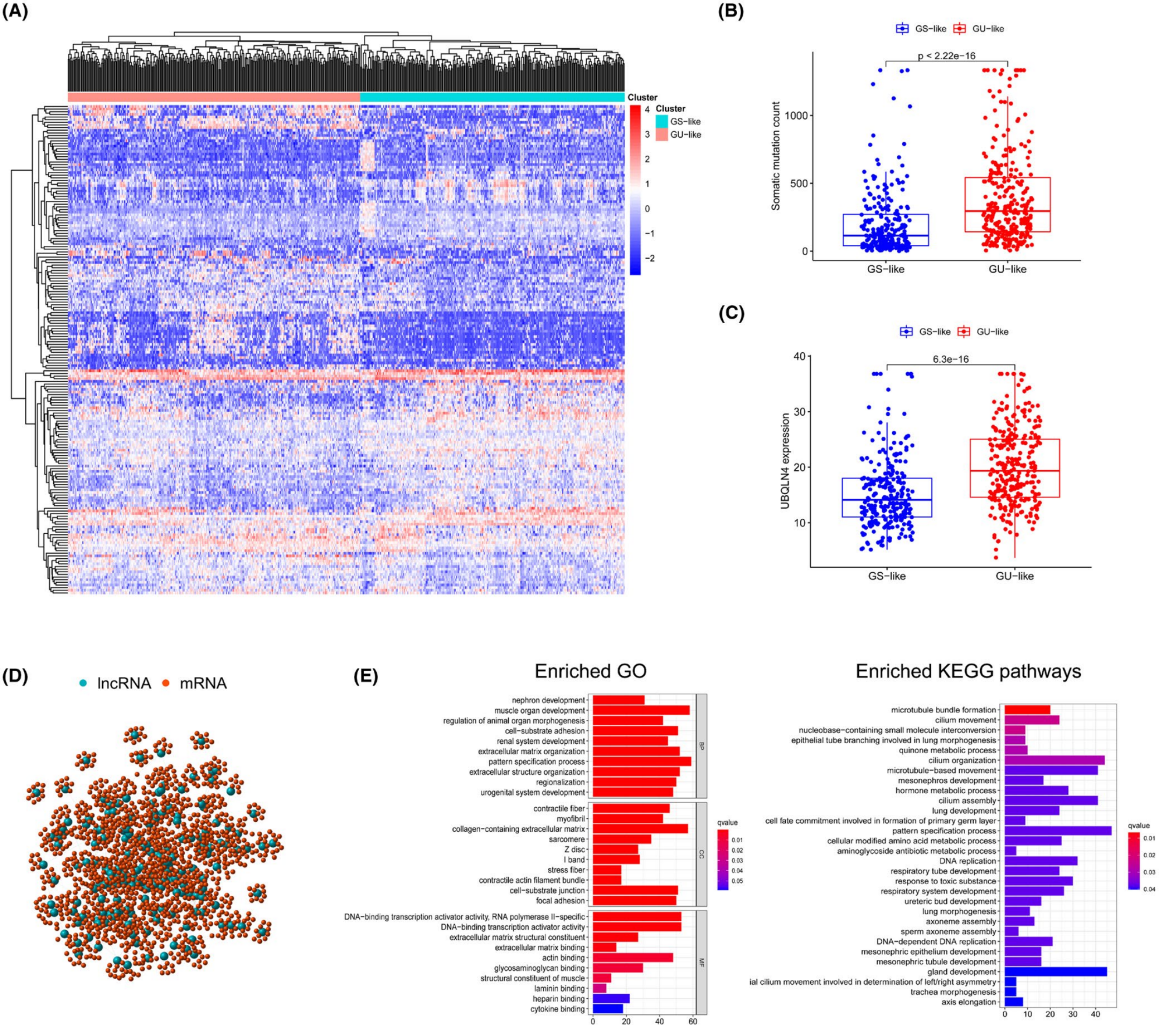
Identification of lncRNAs linked to genomic instability in LUAD patients and further functional enrichment analysis. (A) Unsupervised clustering dependent on the 185 selected genomic instability‐related lncRNAs’ expression trend in 490 LUAD patients. The left red cluster represents the GU‐like group, while the GS‐like group is reflected by blue cluster on the right. (B) Boxplots of somatic mutation counts. The total number of somatic mutations was markedly different between the two groups. For statistical study, the Mann‐Whitney *U* test was used. Median values were reflected by the horizontal points. (C) Boxplots of the UBQLN4 level in both groups. The UBQLN4 level was obviously lower in the GS‐like group than in the other group. (D) Co‐expression network on the basis of the Pearson correlation coefficient analysis of genomic instability‐associated lncRNAs and mRNAs. LncRNAs are described by red circles, and mRNAs are represented by blue circles. (E) GO and KEGG functional enrichment study for co‐expressed mRNAs

In order to classify lncRNAs which might be closely linked to genomic instability, we estimated the total count of somatic mutations in each LUAD patient. The numbers were then sorted by declining order. Then, the last quarter of patients (*n* = 139) and the top quarter of patients (*n* = 134) were selected as the GS‐like group and GU‐like group separately. Comparison of the expression profiles of lncRNAs between the two groups was conducted to classify which lncRNAs were significantly distinct. Through SAM method, 185 lncRNAs were then identified, whose expression levels were signicantly different. The fold change value of these lncRNAs was less than −1.0 or greater than 1.0, and the *p*‐value adjusted by FDR was less than 0.05. Among them, 80lncRNAs were found upregulated in GU‐like group, while other 105 ones were downregulated (Table S1). Analysis of hierarchical clustering was carried out on the LUAD samples from the TCGA database using the lncRNAs extracted from the analysis of genes differentially expressed between groups. Samples were separated into two groups based on the expression of 185 selected lncRNAs (Figure 2A). In the two groups, the pattern of somatic mutation was markedly different. The group that had higher somatic mutations count was designated as the GU‐like group, while the other group was designated as the GS‐like group. The median level of somatic mutations was substantially higher in the GU‐like group than in the GS‐like group (*p *< 0.001, *U* test; Figure 2B). By comparing the expression levels of the UBQLN4 between the two groups, we found that the expression level of UBQLN4 was far higher in the GU‐like group than in the GS‐like group (*p* < 0.001, Mann–Whitney *U* test).

Functional enrichment review was conducted to decide which functions any of the 185 lncRNAs might have, and determine whether their functions were correlated with genomic instability. We quantified the relationship between the 185 lncRNAs and protein‐coding genes (PCGs), and picked the top 10 PCGs that displayed the highest correlation with each lncRNA. We built a set of RNA‐mRNA co‐expression network, the nodes in which represented lncRNA and mRNAs. As shown in Figure 2D, the lncRNA and mRNA were connected if they were related to each other. In light of GO analysis shown in Figure 2E, mRNAs involved in this co‐expression network had a close relationship with the creation of genomic instability, since they were linked to DNA binding transcription activator activity, and actin binding and RNA polymerase II‐specific DNA‐binding transcription activator activity. KEGG pathway studies on PCGs correlated with lncRNAs also revealed 30 substantially enriched pathways, several main pathways of which are correlated with genomic instability, like DNA replication, nucleobase‐containing small molecule interconversion and DNA‐dependent DNA replication.

### 3.2 Development of GILncSig for predicting the clinical outcome in training set

All of those 490 TCGA patients with LUAD were randomly separated into two sets, named the training set (*n* = 246) and the testing set (*n* = 244), to explore the prognostic roles of these GILncRNAs. To classify prognostic‐related lncRNA, we assessed the association between lncRNAs expression levels and OS using Cox regression analysis in the training sample. There were ten hub candidate GILncRNAs in the analysis, and they showed a closely correlation with the outcomes of LUAD patients (*p* < 0.05; Table 2). Multivariate Cox proportional hazard regression analysis was then carried out among the ten candidate lncRNAs and common clinical features (such as age, sex, and stage) to decide if those candidate lncRNAs could serve as independent prognostic factors. Finally, six of ten selected lncRNAs (RHOXF1‐AS1, PLAC4, LINC01116, AC099850.4, LINC01671 and FAM83A‐AS1) endured the multivariate checking for significance and preserved their prognostic significance (Table S2). Based on expression levels of six GILncRNAs, a GILncSig score was established to accurately evaluate the risk of LUAD patients as follow: GILncSig score = (−0.2206 × expression level of RHOXF1‐AS1) + (0.0266 × expression level of PLAC4) + (0.1233 × expression level of LINC01116) + (0.0473 × expression level of AC099850.4) + (0.0558 × expression level of LINC01671) + (0.0316 × expression level of FAM83A‐AS1).

**TABLE 2:** Univariate Cox regression study of 10 of 185 lncRNAs correlated with genomic instability linked with overall survival in LUAD

Ensembl ID	Gene symbol	Genomic location	HR	95% CI	*p*‐value
ENSG00000258545	RHOXF1‐AS1	chrX:120,036,236‐120,146,855	0.767	0.608–0.967	0.025
ENSG00000273877	AC236972.3	chrX:153,225,649‐153,230,357	0.715	0.516–0.989	0.0427
ENSG00000280109	PLAC4	chr21:41,175,231‐41,186,788	1.033	1.011–1.056	0.003
ENSG00000163364	LINC01116	chr2:176,625,118‐176,638,186	1.152	1.101–1.206	<0.001
ENSG00000265415	AC099850.4	chr17:59,202,677‐59,203,829	1.064	1.026–1.103	0.001
ENSG00000251026	LINC02163	chr5:104,079,847‐104,406,121	1.234	1.010–1.508	0.039
ENSG00000225431	LINC01671	chr21:42,579,759‐42,615,095	1.039	1.011–1.068	0.005
ENSG00000262454	MIR193BHG	chr16:14,301,364‐14,336,038	1.134	1.007–1.277	0.038
ENSG00000232415	ELN‐AS1	chr7:74,048,744‐74,062,301	0.909	0.834–0.990	0.029
ENSG00000204949	FAM83A‐AS1	chr8:123,193,507‐123,202,744	1.036	1.006–1.066	0.016

**FIGURE 3:**
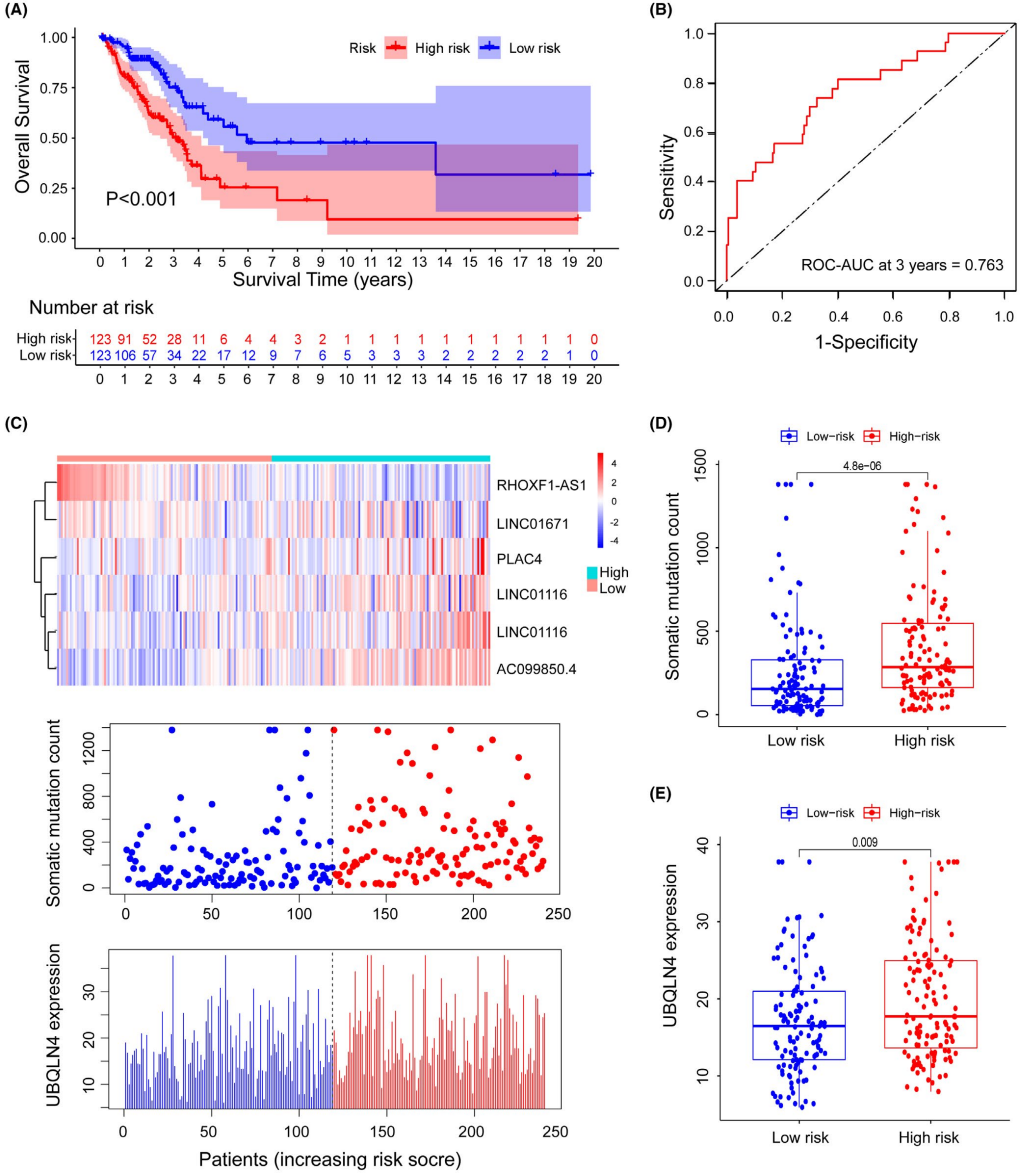
Identification of the lncRNA signature derived from genomic instability for predicting the LUAD patients' clinical outcomes. (A) Estimates of the LUAD patients' overall survival (OS) in the training set using the Kaplan‐Meier method. All 246 LUAD patients in this set were assigned into the high‐risk or the low‐risk group. Univariate Cox analysis and the log‐rank test were carried out to do the statistical analysis. (B) Analysis of the 3‐year time‐dependent ROC curves for the signature in the training set. (C) LncRNA expression patterns, somatic mutation number's distribution and the UBQLN4 expression's distribution along with the increase of the GILncSig scores. (D) Distribution of somatic mutations for LUAD patients in two risk groups. (E) Expression of UBQLN4 in both groups with different risks. Median values were reflected by horizontal lines. The Mann‐Whitney *U* test was carried out to analyze those statistics

The coefficients of lncRNA PLAC4, LINC01116, AC099850.4, LINC01671 and FAM83A‐AS1 were positive in the GILncSig, suggesting that they may be risky factors since their high expressions were linked to bad prognoses. Meanwhile, lncRNA RHOXF1‐AS1 had a beneficial effect since the high expression of it was linked to longer survival. Based on the signature associated with genomic instability, we measured each patient's risk score, the median one of which was utilized to evaluate the cutoff scores for dividing the LUAD patients into two separate prognostic classes. Patients whose scores are higher than or equivalent to the cut‐off were deemed high‐risk, while the majority of patients were categorized as low‐risk. There was a marked difference in the survival rates between patients of the two risk groups (*p *< 0.001, log‐rank test). The low‐risk group at 3 years had a survival rate of 27.6 percent, much higher than that of the high‐risk group (22.8%; Figure 3A). The 3‐year area under curve (AUC) built from the time‐dependent ROC curve in the training set was approximately 0.763 (Figure 3B). UBQLN4 (Ubiquilin 4) is a regulator of protein degradation,^33^ and it mediates the proteasome targeting of misfolded or mislocated proteins.^34^ UBQLN4 is also reported as a key regulator that inhibits homologous recombination repair, thereby promoting genomic instability.^35^, ^36^ The patients were sorted according to their ranking, to observe how the expression of UBQLN4, the total count of somatic mutations and the level of the lncRNAs involved in the GILncSig shift together with the rise of the score (Figure 3C). In patients with poorer scores, the expression levels of AC099850.4, LINC01116, PLAC4, LINC01671 and FAM83A‐AS1 were also low, whereas the expression for RHOXF1‐AS1 was relatively high. However, in the patients with higher scores, the expression levels of the six lncRNAs displayed opposite performances. The findings of the comparison of UBQLN4 expression and somatic mutation analysis revealed significantly difference between the two groups. The count of somatic mutations in LUAD patients was notably lower in the low‐risk group than in the other risk group (*p* < 0.001, *U* test; Figure 3D). Also, Figure 3E also revealed that the expression level of UBQLN4 was meaningfully lower in the group with low risk than in the group with high risk (*p* = 0.009, Mann–Whitney *U* test).

### 3.3 Further validation of GILncSig in the testing set and the TCGA set

**FIGURE 4:**
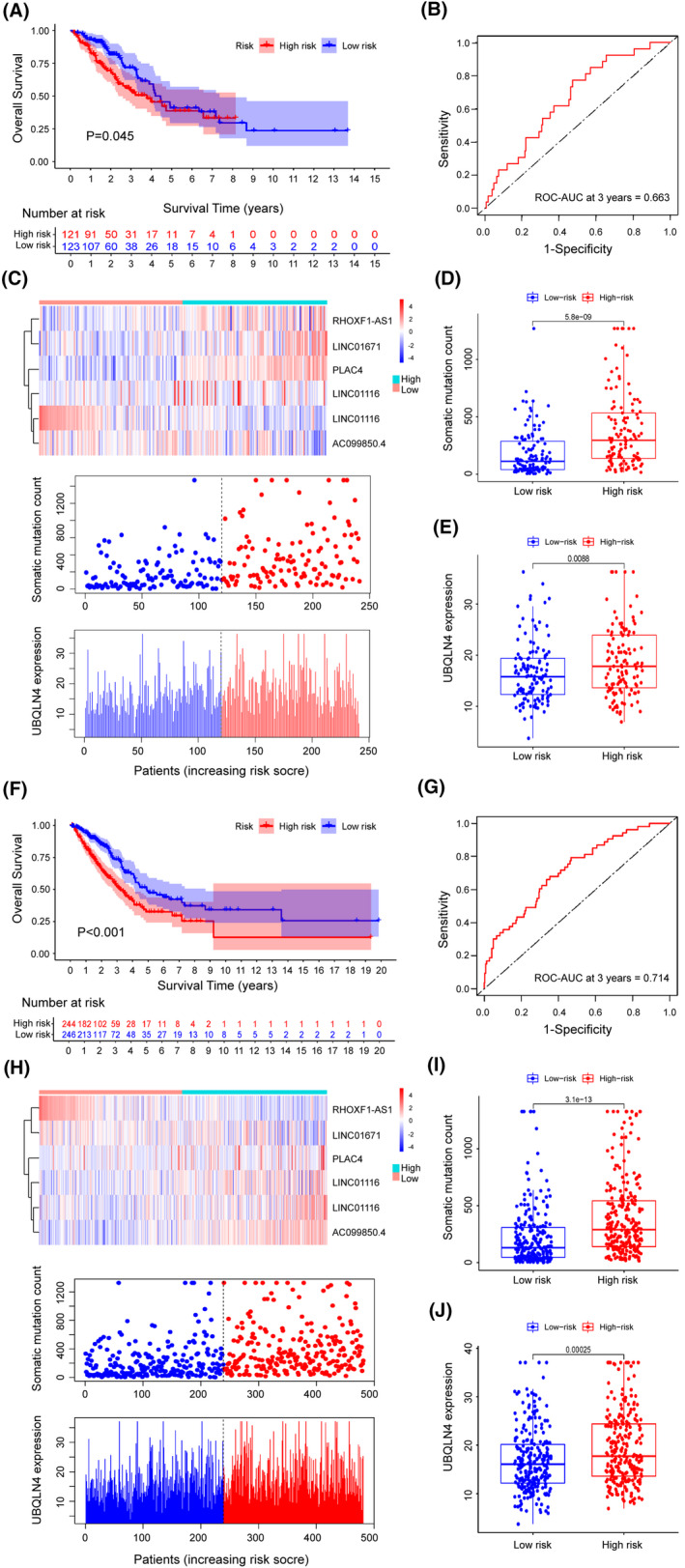
Evaluation of the GILncSig performance in the testing and the TCGA sets. (A) Estimates of OS by Kaplan‐Meier in the testing set for low‐ or high‐risk patients. (B) Analysis of ROC curves dependent on time and AUC for 3‐year OS in the testing set for the GILncSig. (C) LncRNA expression patterns, somatic mutation number's distribution and the UBQLN4 expression's distribution for LUAD samples in the testing set. (D) Distribution of somatic mutations for patients with LUAD in two risk groups of the testing set. (E) UBQLN4 expression levels in the two groups with different risks. (F) Kaplan–Meier estimates of OS of two risk groups in the TCGA set. Analysis of statistics was carried out with univariate Cox regression and the log‐rank test. (G) Analysis of ROC curves dependent on time and AUC for 3‐year OS in the whole TCGA set. (H) LncRNA expression patterns, somatic mutation number's distribution and the UBQLN4 expression's distribution for samples in the TCGA set. (I) Distribution of somatic mutations in two risk groups of samples from the TCGA set. (J) UBQLN4 levels in the two groups in the TCGA set. Median values were reflected by the horizontal points. The Mann‐Whitney *U* test was carried out to complete the statistical analysis

We applied the GILncSig to the other separate testing set with 244 patients and checked the precision of its predictive potential. When the same GILncSig and corresponding cutoff were added to the testing set, the patients with LUAD were divided into the high‐risk group and the low‐risk group, which demonstrated distinctly different OS. The OS of samples was statistically higher in the low‐risk group than in the other risk group (*p *= 0.045, log‐rank test; Figure 4A). Additionally, 3‐year survival rate was at 30.8 percent in the low‐risk group, while it was much slower as 25.6 percent in the group with high risk. Figure 4B showed that the 3‐year AUC calculation in the testing set yields an overall performance of 0.663. The count of somatic mutation and the expression degree of UBQLN4 in the research sample is seen in Figure 4C. Further analysis revealed just like the identical findings in the training set above, the counts of somatic mutations were greatly different between the two risk groups of the testing set (*p *< 0.001, Mann–Whitney *U* test; Figure 4D). Besides, Figure 4E showed that the UBQLN4 expression level was considerably lower in the low‐risk group than in the high‐risk group (*p *< 0.001, Mann–Whitney *U* test).

In the TCGA set, all results obtained from the prognostic analysis of the GILncSig showed similar trend. All 490 patients with LUAD in the TCGA set were assigned to either the high‐risk group or the low‐risk group, which separately consisted of 244 and 246 samples. As shown in Figure 4F, the median OS was significantly longer in the low‐risk group than in the other group (*p *< 0.001, log‐rank test). In addition, the 3‐year survival rate in the group with low risk was significantly higher than that in the group with high risk (29.3% vs 24.2%). No major difference was found among the results of the three sets (the training, the testing and the TCGA sets) when the time‐based ROC study was also applied to the TCGA set (Figure 4G). The situation of the expression of six lncRNAs involved in the GILncSig, the distribution of somatic mutation numbers and that of the UBQLN4 expression level were showed in Figure 4H along with the increase of the signature. The high‐risk group had higher mutation rates than the other risk group (*p *< 0.001, Mann–Whitney *U* test; Figure 4I). Moreover, as shown in Figure 4J, the UBQLN4 expression level was meaningfully lower in the low‐risk group than in the group with high risk (*p* < 0.01, Mann–Whitney *U* test).

To further verify associated signaling pathways activated in the high‐risk group, we carried out GSEA analysis based on the TCGA set. As shown in Figure S1, gene sets, which were differentially enriched in the high‐risk group of the TCGA database, were related to genomic instability, such as homologous recombination, cell cycle, mismatch repair, nucleotide excision repair (NER), RNA degradation and P53 signaling pathway.

### 3.4 Further confirmation for applying the lncRNA signature associated with genomic instability to three external LUAD data sets.

**FIGURE 5:**
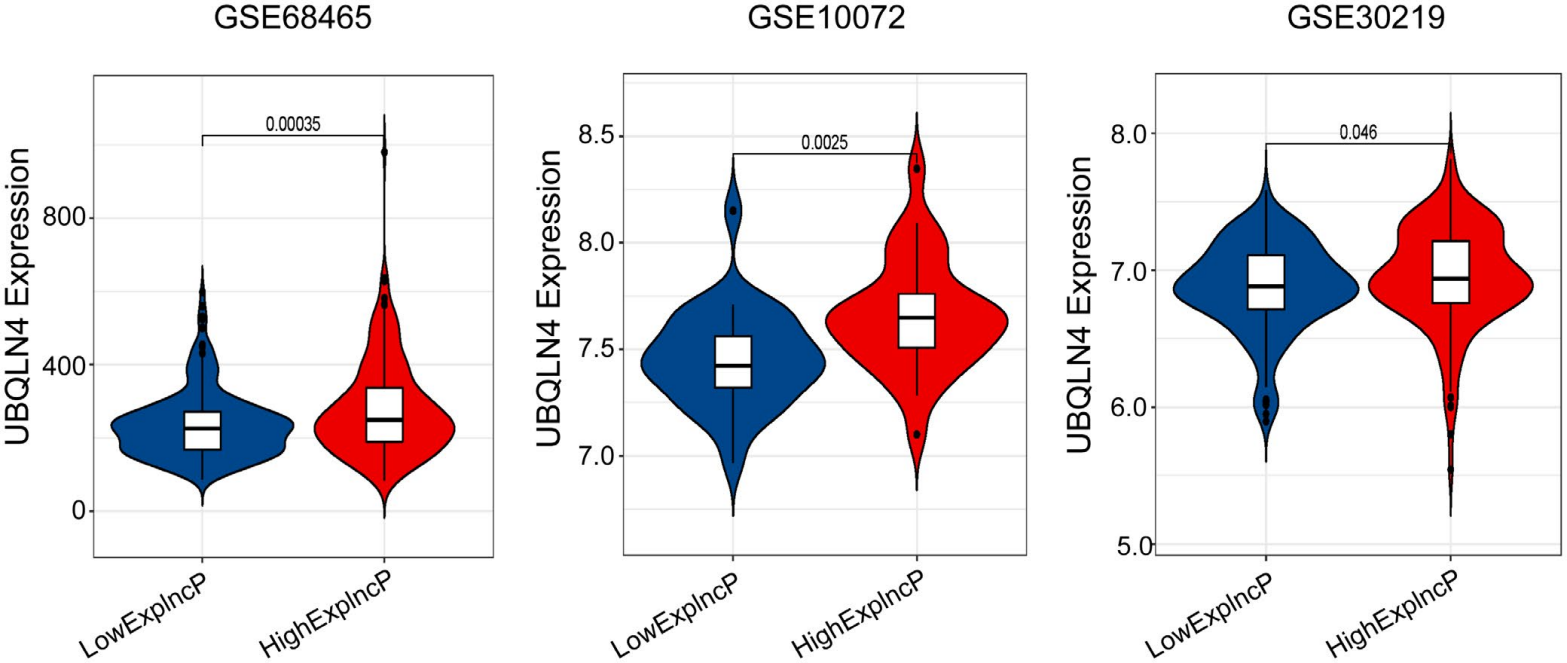
Evaluation of the GILncSig performance in other three separate GEO data sets. Violin plots for the expression levels of UBQLN4 among patients with low and high PLAC4 expression. The Mann‐Whitney *U* test was conducted to compare that between two different risk groups

In order to verify the prognostic value of the GILncSig by applying it to other separate data sets from other platforms, the common microarray array was reannotated. We observed that only one lncRNA, PLAC4, in the six GILncRNAs was covered by GSE68465, GSE10072 and GSE30219, which had large sample sizes and common clinical features. Based on this, to further examine the relation between PLAC4 and LUAD or genomic instability, we explored the relation between the PLAC4 expression and UBQLN4 expression in these three independent data sets. Using Mann–Whitney U test, the violin plots in Figure 5 indicated that the expression levels of UBQLN4 were considerably higher in patients with elevated PLAC4 than in those with low PLAC4 in the three GEO data sets (GSE68465 *p *< 0.001, GSE10072 *p *< 0.01 and GSE30219 *p *< 0.05). These findings were close to the corresponding results found in the sets from the TCGA.

### 3.5 Comparison of efficiency in survival prediction between the GILncSign and existing lncRNA‐dependent signatures in LUAD

**FIGURE 6:**
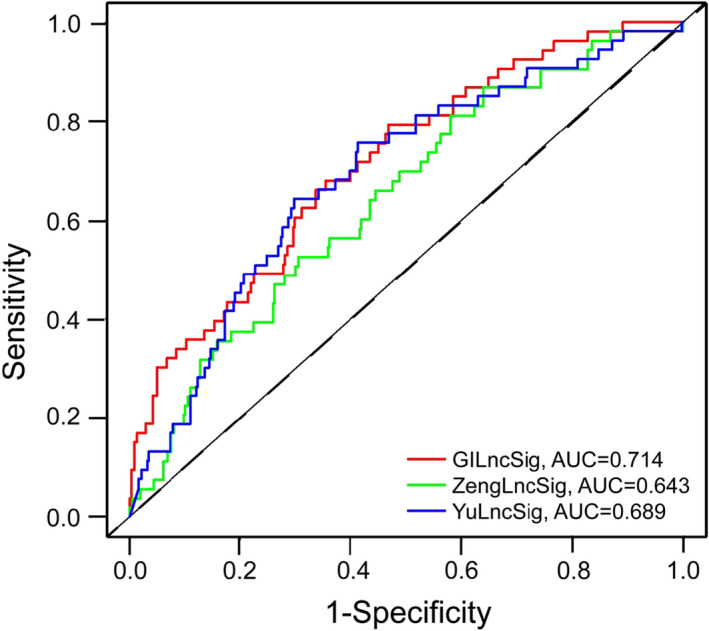
Time‐dependent ROC curves study of 3‐year OS for the GILncSig, YulncSig and ZenglncSig

To further validate the efficiency of the GILncSig in predicting the survival, two signatures based on lncRNAs reported recently were compared with GILncSig in the same TCGA LUAD patient cohort: 2‐lncRNA signature derived from Yu's research (YulncSig),^37^ and 5‐lncRNA signature derived from Zeng's study (ZenglncSig).^38^ It was obvious in Figure 6 that the GILncSig had an AUC value of 0.714 at three years, greater than that of the ZengLncSig (AUC = 0.643) and the YuLncSig (AUC = 0.689). We further performed the time‐dependent ROC curves study of 3‐year OS to compare the predictive efficiency among the GILncSig and two another lncRNA‐based signature in LUAD. The four‐lncRNA signature derived from Shukla's research (ShuklaLncSig) was built based on the analyses of the RNA‐Seq information from TCGA‐LUAD.^39^ While, the six‐lncRNA signature from Miao's study was constructed by analyzing the immune infiltration‐associated lncRNAs in LUAD patients.^40^ As shown in Figure S2, GILncSig had higher AUC value (AUC = 0.714) than that of the MiaoLncSig (AUC = 0.701) and the ShuklaLncSig (AUC = 0.682). The findings suggested the GILncSig had a good predictive performance.

### 3.6 Independence of the established GILncSig from clinical characteristics

**FIGURE 7:**
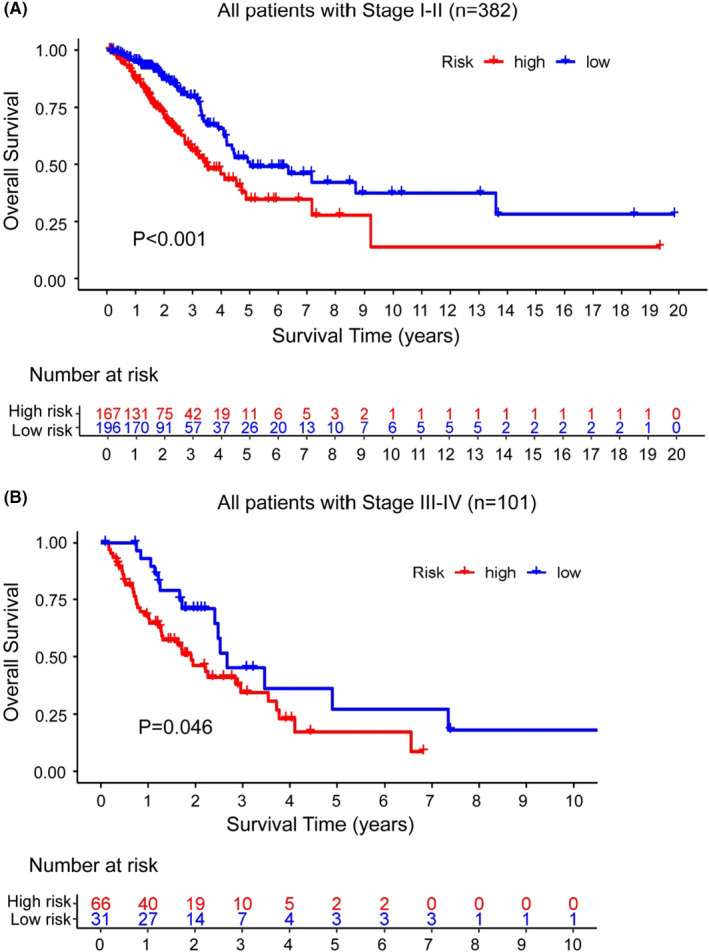
Analyses of stratification by stage. Analyses of OS of two groups with different risks in the early‐stage group (A) and the late‐stage group (B) using Kaplan–Meier curve method. The log‐rank test and univariate Cox analysis were used to do the statistical analysis

Multivariate and Univariate Cox regression analyses were carried out on three common clinical characteristics and the prognostic risk score model, which was based on the GILncSig, to determine if the prognostic significance of GILncSig was independent of other typical clinical factors. As shown in Table 3, after adjusted for the three clinical features, the GILncSig showed a significant correlation with the OS of samples in three sets from TCGA. It was notable in the multivariate Cox analysis that apart from the GILncSig, stage, one of the common clinical characteristics, was also observed significantly correlated to OS. While the distribution of age and gender showed no statistical difference (*p* > 0.05, Figure S3). To determine whether the GILncSig had an independent prognostic value from the stage, we stratified those LUAD patients obtained from the TCGA set by stage. Patients were also classified into two groups; one that was early‐stage and one that was late‐stage. There were 363 LUAD samples in stage I or II involved in the early‐stage group, and the other 97 samples in stage III or IV were involved in the late‐stage group. In early‐stage group, the GILncSig was then applied to separate the 363 samples into two groups, including the high‐risk group (*n* = 167) and the low‐risk group (*n* = 196). Figure 7A showed that the OS was shorter in the high‐risk group of the early‐stage group than in the other group (*p *< 0.001, log‐rank test). Similarly, patients in late‐stage group were separated into low‐risk and high‐risk groups. Between the two groups, the OS was also obviously different (*p* = 0.046, log‐rank test; Figure 7B). Thus, it was apparent that the GILncSig was an autonomous element of prognostic meaning and being correlated with OS. To further confirm the independence of GILncSig, we carried out survival analyses on patients with different stages. As shown in Figure S4, the OS of LUAD patients with high risk in stage I was significantly shorter, so was in high‐risk patients in stage II (*p *< 0.05). However, the results of the OS analyses were not statistically significant (*p *> 0.05), which might be due to the small sample sizes.

**TABLE 3:** Analyses of the GILncSig by Univariate and Multivariate Cox regression models in the three LUAD sets from TCGA

Variables		Univariable model			Multivariable model		
		HR	95% CI	*p*‐value	HR	95% CI	*p*‐value
Training set (*n *= 246)							
GILncSig	High/low	1.037	1.019–1.055	<0.001	1.033	1.015–1.051	<0.001
Age	>65/<=65	1.001	0.981–1.022	0.906			
Gender	Male/female	1.268	0.826–1.946	0.277			
Stage	(III+IV)/(I+II)	1.816	1.473–2.239	<0.001	1.778	1.439–2.197	<0.001
Testing set (*n* = 244)							
GILncSig	High/low	1.073	1.019–1.128	0.007	1.057	1.004–1.112	0.034
Age	>65/<=65	1.007	0.984–1.031	0.544			
Gender	Male/female	0.941	0.616–1.439	0.780			
Stage	(III+IV)/(I+II)	1.500	1.238–1.817	<0.001	1.481	1.219–1.799	<0.001
TCGA set (*n* = 490)							
GILncSig	High/low	1.029	1.019–1.039	<0.001	1.026	1.016–1.036	<0.001
Age	>65/<=65	1.005	0.989–1.020	0.553			
Gender	Male/female	1.113	0.825–1.500	0.484			
Stage	(III+IV)/(I+II)	1.641	1.425–1.890	<0.001	1.623	1.408–1.871	<0.001

## 4 DISCUSSION

Many attempts have been made to investigate the incidence, growth, diagnosis and treatment of LUAD.^41^, ^42^, ^43^, ^44^ Common histopathological features of stage, tumor size and Karnofsky Performance Status (KPS) are now still considered to be the most important prognostic factors in LUAD. Based on their pathological characteristics, LUAD patients are grouped into different groups to accept the corresponding care regimens.^6^, ^45^, ^46^, ^47^, ^48^ However, the clinical performance of patients with LUAD remains somewhat heterogeneous owing to the shortcomings of typical histopathologic characteristics.^49^ Genomic instability has been reported in recent years as the pervasive hallmark of most cancers,^50^, ^51^, ^52^ and is also considered one of the prognostic factors of LUAD.^53^, ^54^ During the development and recurrence of cancers, the diagnostic and prognostic implications of the genomic instability is non‐ignorable.^55^ However, it is a major challenge for us to quantitatively measure the degree of genomic instability. Emerging data indicates that epigenetic and transcriptomic aberrations are closely related to genomic instability.^56^ The identification of PCGs and microRNAs linked to genomic instability has become a top priority. For the prediction of genomic instability, many efforts have also been made to develop miRNA or gene signature.^55^, ^57^

LncRNAs have been considered an important part of tumor biology in recent years. The dysregulated expression of lncRNAs in cancer is associated with the progression of disease. So, they may be potential prognostic markers of cancer.^58^, ^59^, ^60^ Advances in the functional mechanism of lncRNAs make us realize that lncRNAs are also vital to maintaining genome stability, such as NORAD,^61^ H19^62^ and GUARDIN.^63^ For now, the identification of lncRNAs linked to genomic instability and the investigation of their clinical importance are in infancy. In this study, by combining the expression level of lncRNA and the tumor mutator phenotype, we built a frame to identify lncRNAs linked to genome instability. The total number of somatic mutations often represents the burden of mutations in tumors.^64^ It mainly includes a variety of somatic mutations, such as non‐synonymous mutations, insertion/deletion mutations, and silent mutations.^65^ Experiments have confirmed that the total number of somatic mutations is closely related to clinicopathological characteristics,^66^ and often shows no change at relapse, meaning the number is stable.^67^ In addition, somatic mutations and the genomic instability caused by it may be an important driving factor for the development of chemotherapy resistance in malignant tumors.^68^ Hence, we used the total number of somatic mutations as the index for genomic instability.

Through combining the profiles of lncRNA expression with those of somatic mutation in LUAD, we finally found 185 novel lncRNAs linked to genomic instability. We further carried out functional enrichment analyses on genes co‐expressed with the 185 lncRNAs. The results showed these genes enriched in the DNA replication and DNA‐binding transcription activator activity. DNA replication occurs in all organisms and is the basis of biological genetics. The interference with DNA replication can lead to genomic instability,^69^, ^70^ and DNA replication stress is a source of genomic instability, a distinct characteristic of cancer.^71^ An increasing number of studies have tried to understand the role of DNA‐binding transcription activator activity in transcription‐associated genome instability both in prokaryotes and in eukaryotes.^72^, ^73^ These further confirmed that the 185 lncRNAs differentially expressed in two risk groups were linked to genome instability. The abnormal expression of these 185 lncRNAs might destroy the balance of the PCGs regulatory network to influence the usual gene damage repair pathway, which led to the disturbance of cellular genomic stability. Furthermore, we investigated whether the outcomes of LUAD patients could be predicted by using these genomic instability‐associated lncRNAs, and then established a GILncSig consisting of six lncRNAs related to genomic instability (RHOXF1‐AS1, PLAC4, LINC01116, AC099850.4, LINC01671 and FAM83A‐AS1). GILncSig divided LUAD samples in the training set into two risk groups, which had substantially different survival. It was also validated in the testing set and the entire TCGA set. The GSEA results revealed some pathways, such as homologous recombination, mismatch repair, nucleotide excision repair (NER) and RNA degradation, were highly enriched in the high‐risk group. It has been reported that the role of homologous recombination in promoting genomic instability might contribute to the occurrence and development of tumors,^74^ hence homologous recombination should be tightly regulated in order to avoid genomic instability at mitosis.^75^ Mismatch repair often results in microsatellite instability, which is one of two main mechanisms of genomic instability, and tumor predisposition.^76^ NER, as one of four DNA repair systems, requires strict regulation to avoid genomic instability.^77^ The other gene enrichment pathways, such as RNA degradation, P53 signaling pathway and cell cycle, were also observed associated with genomic instability.^78^, ^79^, ^80^ Additionally, the GILncSig is strongly associated with the phenotype of the tumor mutator and the UBQLN4 expression level in LUAD, both of which are essential markers of genomic instability. Moreover, three external GEO data sets showed similar results. It was apparent that the expression level of PLAC4 was linked to that of UBQLN4 in LUAD patients (Figure 5). Up to now, there have been no studies of the biological roles of these lncRNAs involved in the GILncSig after a diligent literature search. However, we noticed that lncRNA; RHOXF1‐AS1, located in chromosome Xq24, has been found to be involved in the development of diseases caused by genetic variation.^81^ LINC01116 is located in 2q31 and known for promoting the development of cisplatin resistance in LUAD.^82^ FAM83A‐AS1 is located in 8q24, whose high expression was recently found correlated positively with a poor prognosis of LUAD.^83^ LncRNA PLAC4 is located in chromosome 21q22 region, where SNP has been found involved in chromosomal aberration‐related diseases.^84^ LINC01671 is located in 21q22 and AC099850.4 is located in Xq28, both of which have not been studied until now. The results of all this validation in different data sets and data collected from literature suggested that apart from owning a prognostic value, the specific GILncSig is also a genomic instability indicator for patients with LUAD, especially in the early stage LUAD.

Although our research provides valuable insights into the assessment of genomic instability and the prognosis of patients with LUAD, we note some major limitations. Firstly, there are some studies that have indicated the total number of somatic mutations may increase with age, so it might not be the optimal choice as the index for genomic instability. Secondly, the information of LUAD patients in GEO sets did not include somatic mutations, and the clinical information and the lncRNA expression data in the GEO sets was incomplete, where we only found the expression information of lncRNA PLAC4 involved in the signature. Hence, more separate data sets with somatic mutations, more complete the lncNRA expression data and clinical characteristics are needed to further verify the prognostic value of the GILncSig. In addition, the GILncSig was actually built basing on our computational frame derived from mutator hypothesis; thus, more practical studies are needed for experimental biologists to explore the GILncSig's regulatory mechanisms in the maintain of genomic instability.

## 5 CONCLUSION

This research suggested a computational frame derived from the mutator hypothesis to classify lncRNAs associated with genomic instability, which offers a crucial approach for investigating functions of lncRNAs in genomic instability. We developed a GILncSig as an independent marker with prognostic value by combining profiles of lncRNA expression with clinical features of LUAD and somatic mutation profiles. More prospective validation in this study shows that the GILncSig may have significant implications on genomic instability, and have some instructive significance in the customized treatment in LUAD patients.

## CONFLICT OF INTEREST

The authors declare no competing interests.

## AUTHORS' CONTRIBUTIONS

Conceptualization, Y.M., G.Z. and N.L.; data curation, C.G., Y.M. and C.J.; formal analysis, Y.M., J.F., and C.J.; methodology, Y.M. and J.F.; supervision, J.F.; validation, C.G., and Y.M.; writing‐original draft, Y.M.; writing‐review and editing, C.G., Y.M. and J.F.

## ETHICS APPROVAL AND CONSENT TO PARTICIPATE

Not applicable.

## CONSENT FOR PUBLICATION

All listed authors took part actively in the research and read and approved the manuscript submitted.

## Supporting information

Fig S1

Fig S2

Fig S3

Fig S4

Table S1

Table S2

## Data Availability

The datasets generated and analyzed during the current study are available in TCGA (http:/cancergenome.nih.gov/abouttcga) and GEO (https://www.ncbi.nlm.nih.gov/geo).
