# Supplementary figures and images for: Computational detection of a genome instability‐derived lncRNA signature for predicting the clinical outcome of lung adenocarcinoma
Source: Cancer Med. 2021 Dec 5;11(3):864–79. doi: 10.1002/cam4.4471 (PMC8817082; doi:10.1002/cam4.4471)

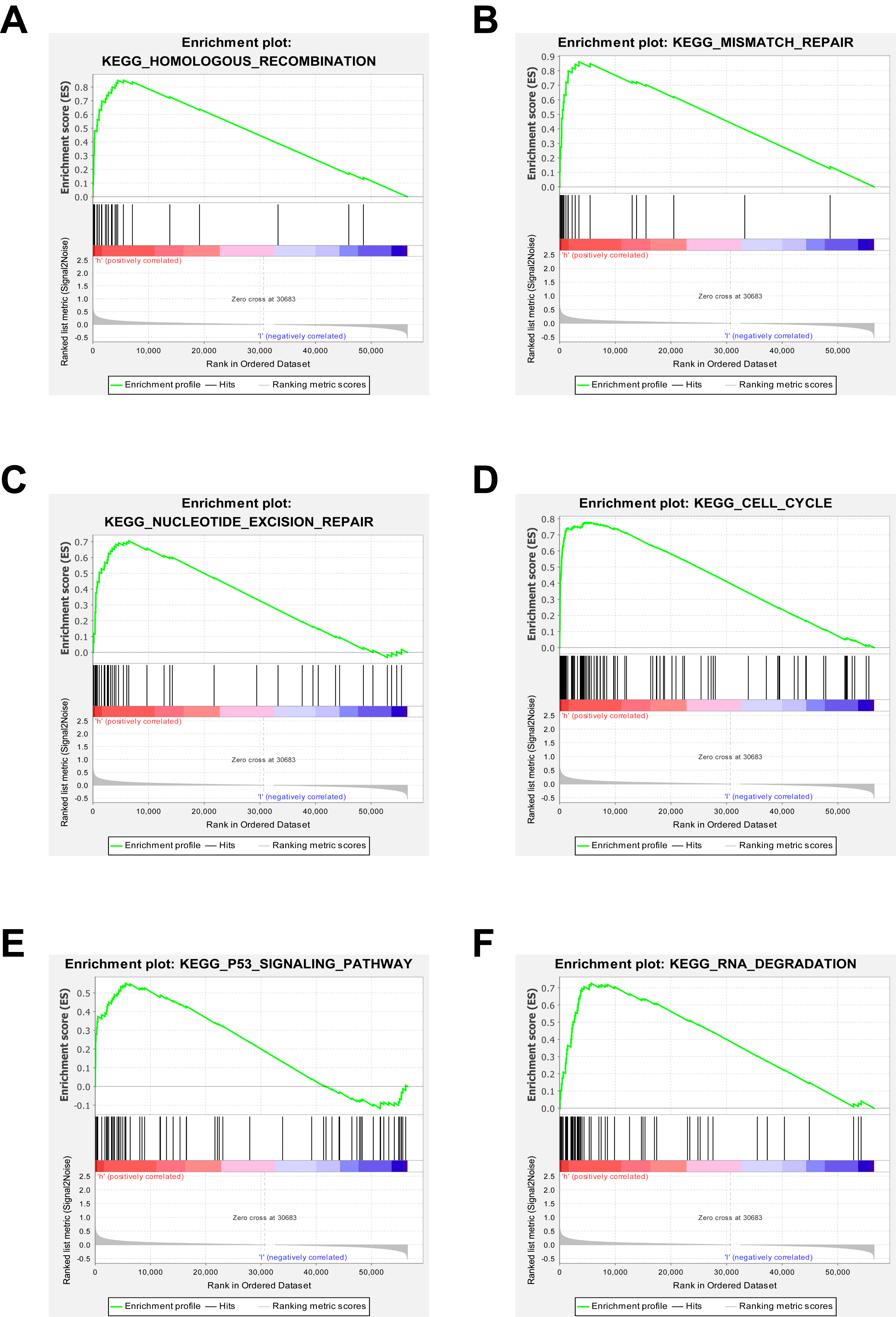

Supplement: Supplementary file 1 — Fig S1 [file CAM4-11-864-s002.tif]

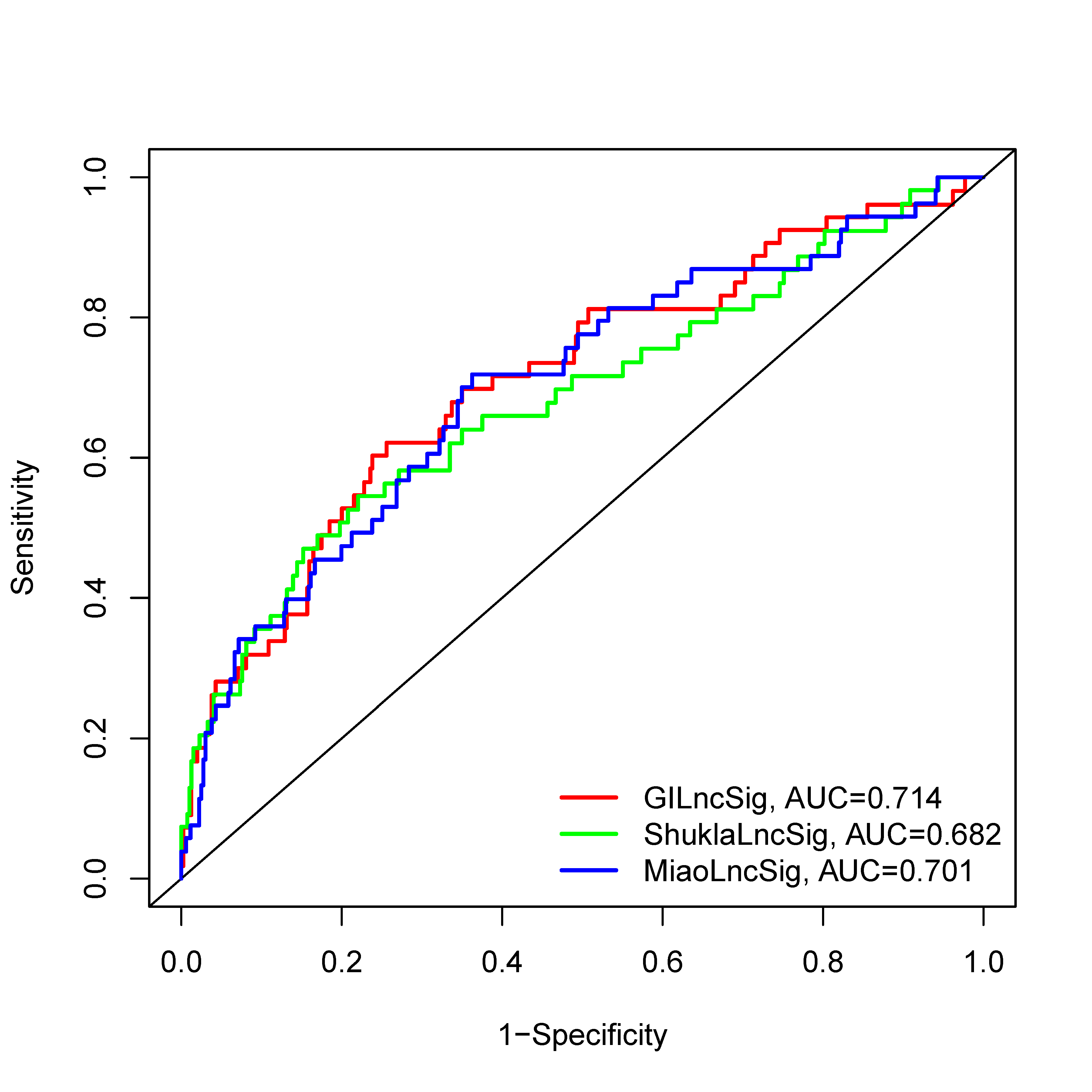

Supplement: Supplementary file 2 — Fig S2 [file CAM4-11-864-s005.tiff]

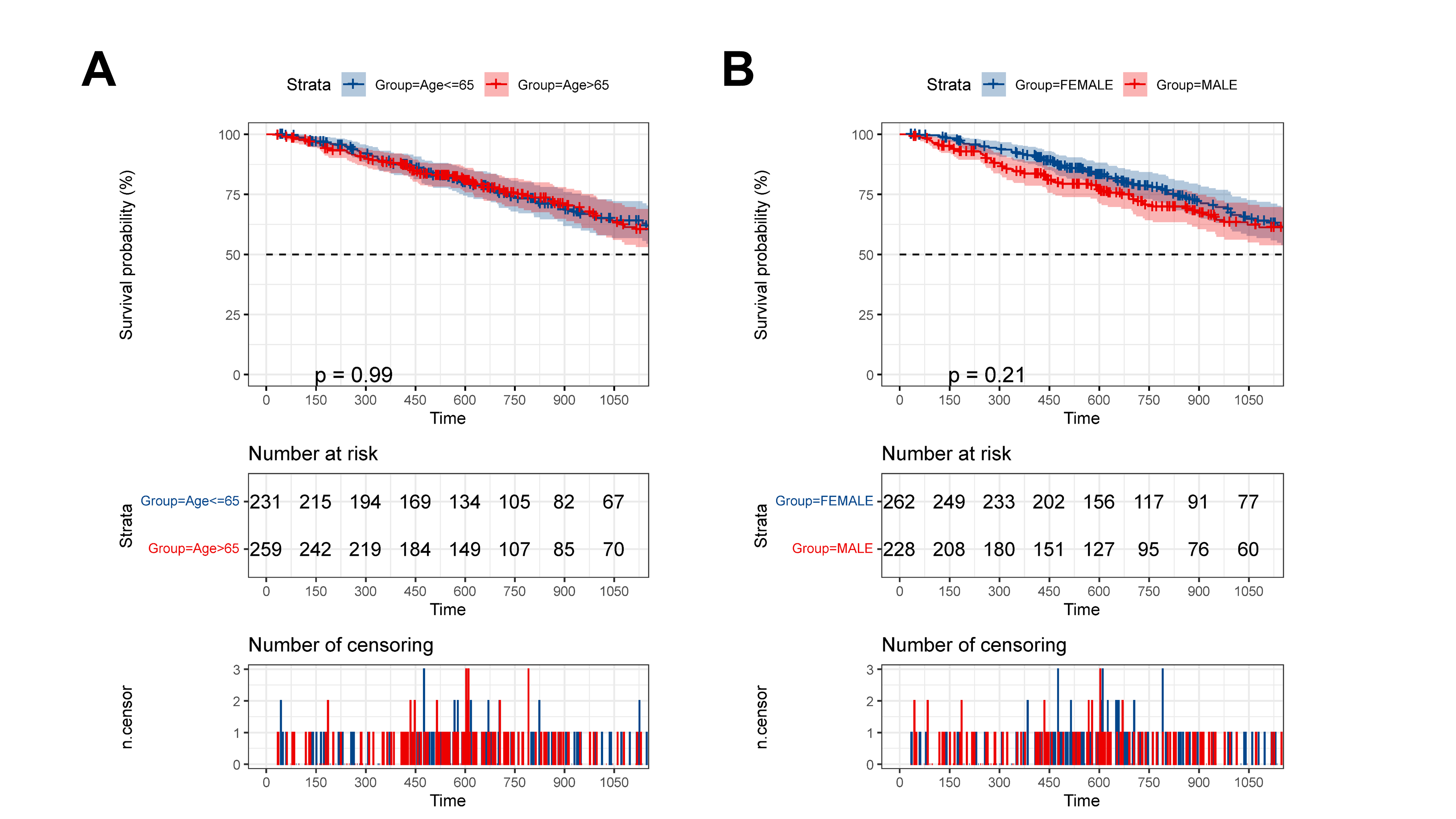

Supplement: Supplementary file 3 — Fig S3 [file CAM4-11-864-s003.tif]

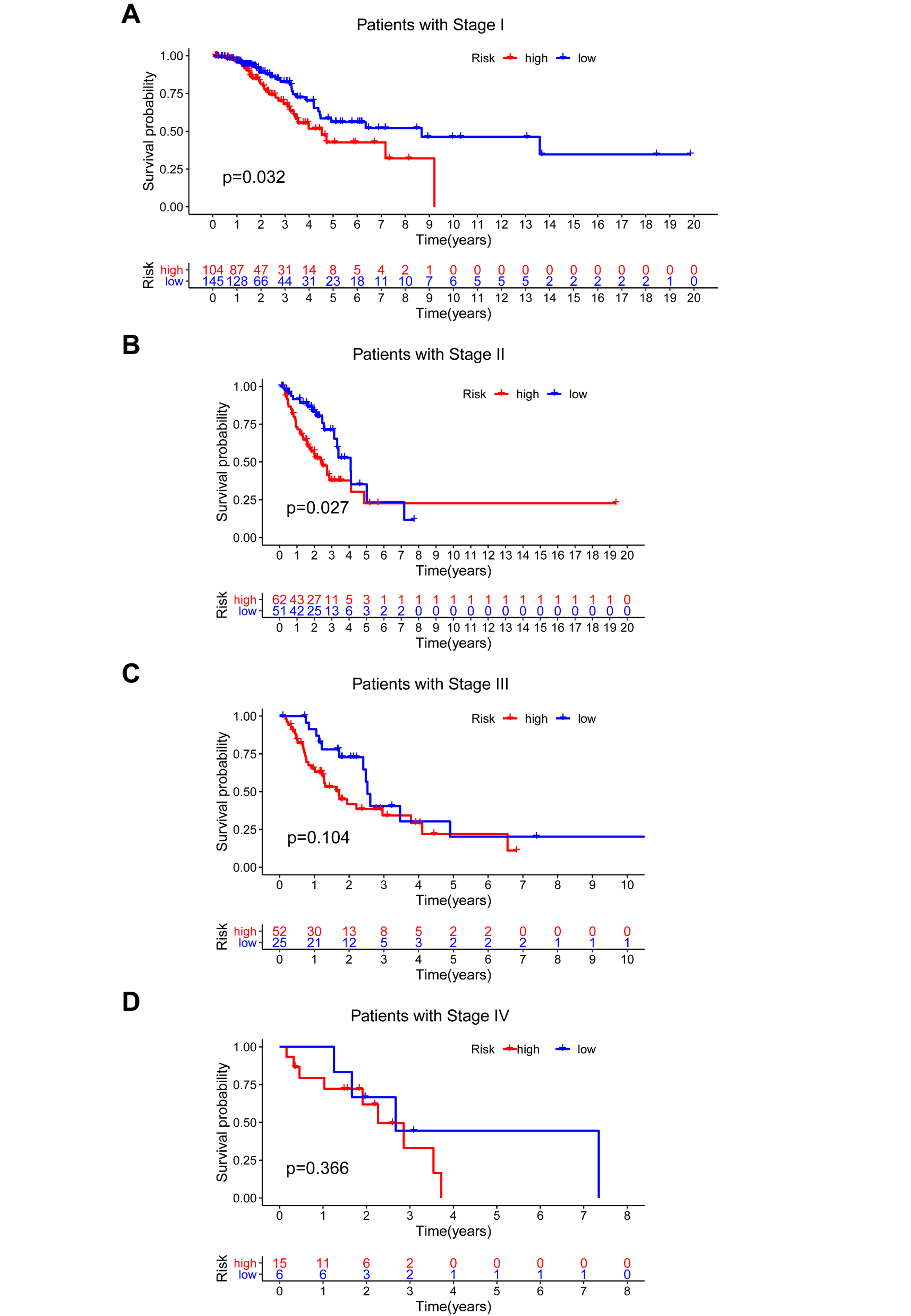

Supplement: Supplementary file 4 — Fig S4 [file CAM4-11-864-s004.tif]
